# Periprosthetic radius fracture after total wrist arthroplasty: A case report

**DOI:** 10.1016/j.tcr.2024.101089

**Published:** 2024-07-29

**Authors:** Ryan Quinn, Matthew Robinson

**Affiliations:** Riverside University Health System, Department of Orthopedics, 26520 Cactus Avenue, Moreno Valley, CA 92555, United States of America

**Keywords:** Wrist arthroplasty fracture distal radius periprosthetic periimplant

## Abstract

Total wrist arthroplasty (TWA) is indicated in select low demand patients with pan-carpal arthritis to decrease pain and preserve motion. Complications of TWA are well described including aseptic loosening, superficial and deep infection, wound issues, component dislocation, stiffness, and both intraoperative and post-operative fracture. With 4th generation implant designs, the incidence of many of these complications have decreased, but these complications remain challenging to address. In particular, sparse literature is available for the treatment of periprosthetic radius fractures after TWA. This report describes the management of an open, periprosthetic distal third radius fracture 18 months after index TWA in a medically complex 53-year-old female with lag screw fixation and a dorsal wrist spanning plate (DSP).

## Introduction

Total wrist arthroplasty (TWA) is indicated in low demand patients with pan-carpal arthritis secondary to post-traumatic arthropathy, advanced inflammatory arthropathy, and, less frequently, primary osteoarthritis [1,2]. TWA provides pain relief and preserves suitable range of motion for activities of daily living (ADLs) in comparison to radiocarpal fusion, and is preferred by many patients [[1], [2], [3]].

TWA complication rates vary from 11 % to 43 %, including superficial wound dehiscence, post-operative stiffness, dislocation, impingement, aseptic loosening, infection, and periprosthetic fracture [1,3]. Periprosthetic fractures are a rare occurrence following total wrist arthroplasty, estimated to occur in 0.4 % to 2 % of TWA cases, with metacarpal fractures being more common [4,5]. A paucity of literature is available on the management of periprosthetic radius fractures. [4,[6], [7], [8]], and no fracture classification exists. As complication rates continue to decrease as TWA implant designs continue to evolve [5], TWA's may soon become a more favorable option in the treatment of wrist arthritis and the prevalence of post-operative periprosthetic fracture will likely increase.

In this report, we describe one method of fixation for a periprosthetic radius fracture following a total wrist arthroplasty with a stable radial component.

## Case report

**Fig. 1:**
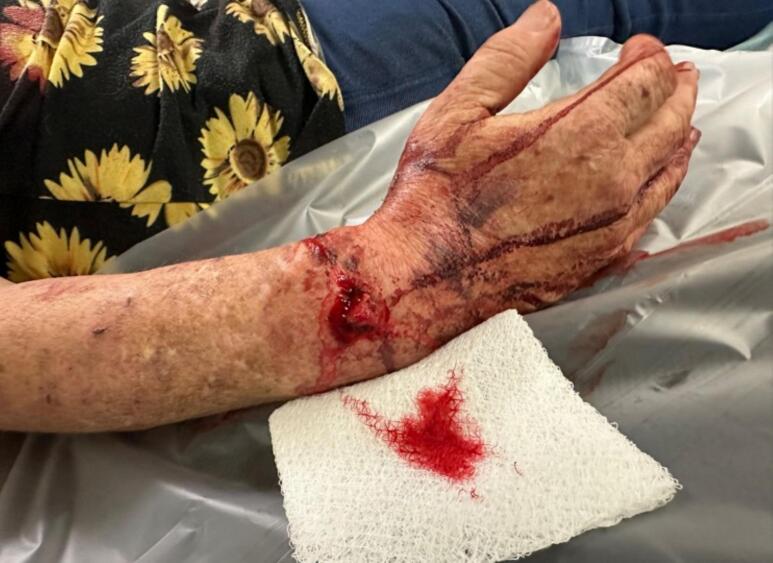
Clinical image on presentation of the right dorsal wrist open fracture wound.

**Fig. 2:**
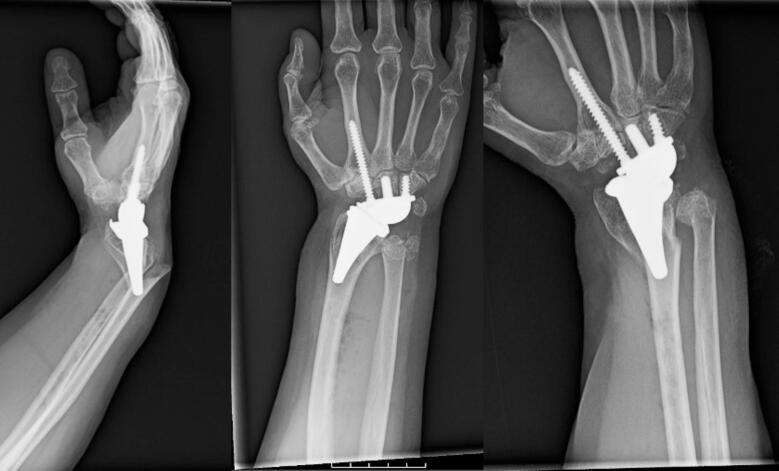
Right wrist radiographs on presentation to the Emergency Department prior to closed reduction demonstrating peri-implant distal 1/3rd radius fracture and distal ulna fracture. Of note, the radial component appears well fixed in the distal bone.

A 53-year-old female presented to our level-1 trauma center with a grade II open, right periprosthetic distal third radius and ulna fracture, and a closed left distal radius fracture, after a ground level fall while sleep walking. The patient had a past medical history of chronic obstructive pulmonary disease (COPD), congestive heart failure (CHF), hepatitis C virus with thrombocytopenia (HCV), daily tobacco use and a right TWA (Kinematx, Extremity Medical, Parsippany, NJ) performed 18 months prior to presentation at an outside institution for right wrist arthritis secondary to Preiser's disease. Examination of the right wrist demonstrated a 2 cm open wound over the dorsal distal radius, with the proximal end of the radial fracture exposed, and intact neurovascular status [Fig. 1]. Radiographs of the right wrist [Fig. 2] and left wrist were obtained.

The patient was treated in the Emergency Department with intravenous antibiotics, bedside wound irrigation of the open fracture wound, and a closed reduction of both the right and left wrist fractures. She was taken to the operating room within 24 h for simultaneous open reduction and internal fixation of the right and left distal radius fractures. The left closed distal radius fracture was treated with a standard volar locking plate using a modified Henry approach without complication.

**Fig. 3:**
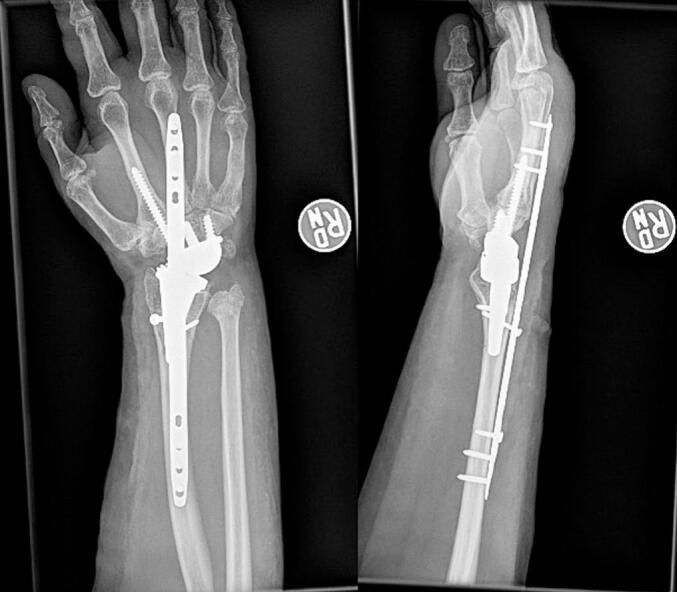
Right wrist radiograph 1 week post-operatively.

Due to the poor bone quality and minimal fixation available at the right periprosthetic distal radius fracture, a dorsal spanning plate (DSP) (Depuy-Synthes, Warsaw, IN) was selected as an internal bridging device to control the lever arm at the fracture site. A dorsal approach to the wrist and forearm utilizing the open fracture wound was used, and an extensive debridement and irrigation as performed. The radial prosthesis was found to be well fixated in the bone distal to the fracture. The fracture margins were debrided, and reduction was obtained using two pointed reduction clamps. A 2.7 mm lag screw was then placed to maintain reduction. The dorsal spanning plate was then affixed to bone using three bicortical 2.4 mm screws in the 3rd metacarpal shaft and three bicortical 2.7 mm screws in the radial shaft. An additional 2.4 mm lag screw was placed last through the plate for additional compression and fixation at the fracture site. The surgical wound was irrigated with normal saline, the surgical wound was closed and the patient was placed in a volar slab splint. Fig. 3 demonstrates radiographs 1 week post-operatively.

**Fig. 4:**
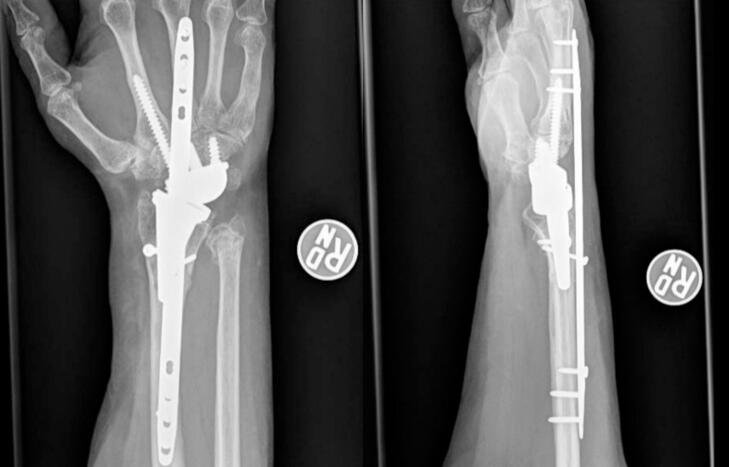
Right wrist radiograph 2.5 months post operatively demonstrating interval healing of radius and ulna fractures.

**Fig. 5:**
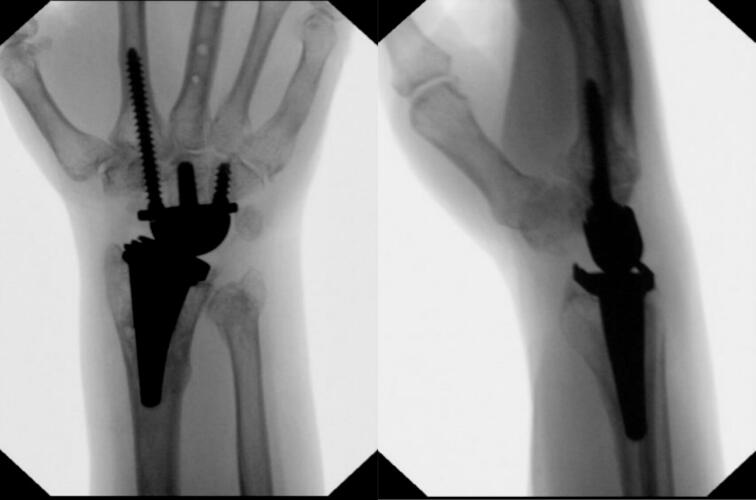
Right wrist intraoperative images demonstrating removed dorsal spanning plate, healed periprosthetic radius fracture, and with stable total wrist arthroplasty components.

The patient desired to follow up with the surgeon who performed her TWA for her post-operative care at an outside institution. At three months, the dorsal spanning bridge plate was removed. Fig. 4 depicts radiographs at 2.5 months post-operatively. Fig. 5 depicts radiographs at the time of hardware removal.

At the patient's 2 week post-operative visit from her hardware removal, she had 30 degrees of active right wrist flexion, 10 degrees of active right wrist extension. At 5 months post-operatively, she had 53 degrees active flexion, 52 degrees active extension, deemed to be functional and adequate for her activities of daily living [9].

## Discussion

Periprosthetic radius fractures after total wrist arthroplasty remain a rare complication, occurring in 0.4 % to 2 % of total wrist arthroplasty cases and literature describing the method and rational of treatment of fractures after TWA remains limited [[4], [5], [6], [7]].

Options based on the current literature for the treatment of periprosthetic radius fractures after TWA include splint or cast immobilization, open reduction internal fixation, revision arthroplasty, resection arthroplasty, and hardware removal with wrist arthrodesis based upon fracture morphology, bone quality, patient characteristics and surgeon comfort. As these fractures are a rare occurrence, no consensus on the best treatment is available, and no fracture classification driving treatment has been proposed. Non-operative treatment should be reserved for stable fractures involving the distal tip of the component, due to the high rate of non-union or mal-union as noted by Dawson. As with the Vancouver classification in periprosthetic fractures of the proximal femur, fractures with a stable radial component and adequate bone stock are amenable to open reduction and internal fixation. Fractures with a loose radial component or inadequate distal radius bone stock require resection arthroplasty, wrist arthrodesis or revision arthroplasty based on the surgeons comfort level and experience.

Options for ORIF include a dorsal or volar based distal radius plate, or a DSP. The use of a dorsal spanning plate (DSP) was originally described by Burke and Singer in 1998 for treatment comminuted, unstable distal radius fractures, as an alternative to external fixation [10]. Further literature then applied the use of a DSP for treatment of comminuted distal radius fractures in the elderly with osteoporotic bone, followed by DSP removal after fracture healing to allow for wrist range of motion. In our case, we used the DSP in this same manner, as an internal external fixator, to protect our lag screw fixation in the setting of osteoporotic bone in a medically complex patient. In our patient, unicortical screws about the radial component affixed with a dorsal radius plate, as used by Barrerra-Ochoa [8], would not have provided enough stability in the setting of osteoporotic bone.

Our case describes one method of treatment of an open, periprosthetic radius fracture after a TWA in which the radial component remained stable in the setting of bilateral wrist fractures. The fracture was treated with debridement and irrigation, anatomic reduction, lag screw fixation, dorsal wrist spanning plate application, and spanning plate removal at 3 months from date of injury. To our knowledge, this is the first case demonstrating retained wrist range of motion after dorsal spanning plate removal in the setting of a total wrist arthroplasty. With our approach, our patient was able to avoid undesirable outcomes including non-union, revision arthroplasty and wrist arthrodesis and was also able to achieve pain free active wrist range of motion sufficient for ADL's after dorsal spanning plate removal.

## Statement of informed consent

The patient provided informed consent for de-identified images and clinical information related to the case to be used for educational and research purposes.

## Disclosures

The authors have no funding or conflict of interest disclosures to address. This work has not been presented at any prior meeting or published in the literature elsewhere.

## CRediT authorship contribution statement

**Ryan Quinn:** Conceptualization, Writing – original draft. **Matthew Robinson:** Conceptualization, Writing – review & editing.

## Declaration of competing interest

The authors declare that they have no financial or personal relationships that could have influenced the work reported in this article. There was no outside funding source.
